# Handling Incomplete or Late-Onset Toxicities in Early-Phase Dose-Finding Clinical Trials: Current Practice and Future Prospects

**DOI:** 10.1200/PO.23.00441

**Published:** 2024-01-05

**Authors:** Zhulin Yin, Adrian P. Mander, Johann S. de Bono, Haiyan Zheng, Christina Yap

**Affiliations:** ^1^ Clinical Trials and Statistics Unit, Institute of Cancer Research, London, United Kingdom; ^2^ Centre for Trials Research, Cardiff University, Cardiff, United Kingdom; ^3^ Drug Development Unit, The Institute of Cancer Research and the Royal Marsden NHS Foundation Trust, London, United Kingdom; ^4^ Department of Mathematical Sciences, University of Bath, Bath, United Kingdom

## Abstract

**PURPOSE:**

The way late-onset toxicities are managed can affect trial outcomes and participant safety. Specifically, participants often might not have completed their entire follow-up period to observe any toxicities before new participants would be recruited. We conducted a methodological review of published early-phase dose-finding clinical trials that used designs accounting for partial and complete toxicity information, aiming to understand (1) how such designs were implemented and reported and (2) if sufficient information was provided to enable the replicability of trial results.

**METHODS:**

Until March 26, 2023, we identified 141 trials using the rolling 6 design, the time-to-event continuous reassessment method (TITE-CRM), the TITE-CRM with cycle information, the TITE Bayesian optimal interval design, the TITE cumulative cohort design, and the rapid enrollment design. Clinical settings, design parameters, practical considerations, and dose-limiting toxicity (DLT) information were extracted from these published trials.

**RESULTS:**

The TITE-CRM (61, 43.3%) and the rolling 6 design (76, 53.9%) were most frequently implemented in practice. Trials using the TITE-CRM had longer DLT assessment windows beyond the first cycle compared with the rolling 6 design (52.5% *v* 6.6%). Most trials implementing the TITE-CRM (91.8%, 56 of 61) failed to describe essential parameters in the protocols or the study result papers. Only five TITE-CRM trials (8.2%, 5 of 61) reported sufficient information to enable replication of the final analysis.

**CONCLUSION:**

When compared with trials using the rolling 6 design, those implementing the TITE-CRM design exhibited notable deficiencies in reporting essential details necessary for reproducibility. Inadequate reporting quality of advanced model-based trial designs hinders their credibility. We provide recommendations that can improve transparency, reproducibility, and accurate interpretation of the results for such designs.

## Teaser

Evaluated late-onset toxicity-focussed dose-finding trials, recommending improved reporting practices

## INTRODUCTION

Many drugs in development, including therapies modulating the immune response and molecularly targeted agents (MTAs), are recognized to have a potential for late-onset toxicities, typically defined as toxicities that occur after the first treatment cycle.^1^ When a new participant is enrolled onto the trial, some of the previous participants might not have reached the end of follow-up, so their dose-limiting toxicity (DLT) information is incomplete. Conventional phase I designs expect DLTs to occur soon after the administration of treatment, usually within the first treatment cycle, and make dose recommendations on the basis of fully observed toxicity data. To account for late-onset toxicities, suspending enrollment until toxicities have been fully captured would mean an extended DLT assessment period and thereby a prolonged trial with increased trial costs. Whereas, ignoring the potential occurrence of late-onset toxicities can lead to underestimation of the toxicity thus result in harmful dose decisions.

The same challenges can arise where the accrual is rapid and continuous. For example, if the accrual rate is one participant every 7 days in a trial with the DLT assessment period of 28 days, when the fifth participant is recruited, the DLT outcomes of the three previous participants could still be pending.

Several designs have been proposed to make interim dose decisions on the basis of partial and complete toxicity information. Such designs can be subdivided into two categories:

1. Model-based or model-assisted approaches, such as the time-to-event continuous reassessment method (TITE-CRM)^2^ and the TITE Bayesian optimal interval design (TITE-BOIN).^3^ Such approaches regard the DLTs as a time-to-event outcome and permit continuous recruitment. These designs have been pursued particularly when the DLT assessment period is long.
2. Algorithm-based approaches, such as the rolling 6 design.^4^ In the rolling 6 design, the dose assigned to a new participant is based on the number of participants with a complete DLT follow-up and the number of participants whose DLT is still pending.

However, the uptake of these designs in clinical trials has been arguably slow.

In this article, we review published early-phase dose-finding trials that have implemented such designs, focusing on how these designs have been conducted and reported. Furthermore, we compare the characteristics of the two most popular designs (ie, TITE-CRM and rolling 6) and retrospectively illustrate their use in two trials to replicate the interim and final analyses. Recommendations are then provided on how to enhance the reporting quality of such designs, including the use of an informative graphic tool to visualize the amount of DLT information (DLT outcome and follow-up time) that contributes to each interim dose decision process.

## METHODS

### Identification of Designs

We searched PubMed for all publications handling late-onset toxicities using the terms “phase I late-onset toxicity” and “phase I delayed toxicity” (November 11, 2020). All relevant phase I designs taking late-onset toxicity into consideration are included. Three additional designs were identified through looking into the publications cited by the phase I designs with consideration of late-onset toxicity. In total, 23 publications met our inclusion criteria (Data Supplement, Fig S1). We then screened publications that have cited these 23 designs to investigate if there are any published trials that have implemented these designs. We identified six designs that have been implemented in published trials, namely, TITE-CRM, TITE-CRM with cycle information consideration,^5^ TITE-BOIN, TITE cumulative cohort design (TITE-CCD),^6^ rolling 6 design, and the rapid enrollment design (RED).^7^ The key features of these designs are summarized in Table 1.

**TABLE 1.**
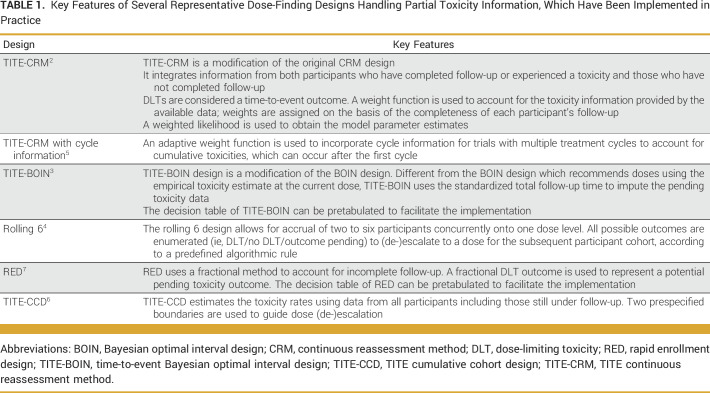
Key Features of Several Representative Dose-Finding Designs Handling Partial Toxicity Information, Which Have Been Implemented in Practice

### Search Strategy for Published Trials

We performed two literature searches: one on April 28, 2021, including all trials published until December 31, 2020, and another expanded search on March 26, 2023, including all trials published between January 1, 2021, and March 26, 2023 (Data Supplement, Fig S2). No restrictions were applied to limit disease types, and only human trials were included. The original articles of the TITE-CRM, TITE-CRM with cycle information, TITE-BOIN, rolling 6, and RED are indexed by PubMed. We screened all publications that have cited these original articles in PubMed. We also screened publications citing another classic TITE-CRM article.^8^ Since the original article for the TITE-CCD is not included in PubMed, we screened the publications that have cited it in Google Scholar instead. Additional keyword search with filters was performed to ensure the completeness of our search results.

1. Filters used: Clinical Study; Clinical Trial; Clinical Trial Protocol; Clinical Trial, Phase I; Clinical Trial, Phase II.
2. Keywords used: For the TITE-CRM design: TITE CRM, time-to-event CRM, time-to-event continual reassessment method.

For the TITE-CRM design with cycle information consideration: TITE CRM, time-to-event CRM, time-to-event continual reassessment method.
For the TITE-BOIN design: time-to-event Bayesian optimal interval design, time-to-event BOIN, TITE BOIN.
For the Rolling 6 design: rolling 6 design, rolling six design.
For the TITE-CCD design: time-to-event cumulative cohort design, TITE CCD
For the RED design: rapid enrollment design

### Data Extraction

We extracted the following information from each article:

1. Trial information: Trial name, therapy type, study population, phase of clinical development, dose-escalation design, published date, starting dose and the number of planned doses, cohort size, sample size (proposed sample size and final sample size).
2. DLT information: Target DLT, DLT follow-up period, total toxicity follow-up period, time to toxicity assumption, time point of a DLT occurrence, DLT redefinition because of late-onset toxicity.
3. Required design parameters: for TITE-CRM: target DLT, weight function, cohort size, skeletons, dose-toxicity model, model parameter prior distribution; for TITE-CCD: prespecified boundaries for dose escalation/de-escalation, target DLT; for TITE-BOIN: prespecified boundaries for dose escalation/de-escalation, target DLT; for RED: target DLT.
4. Practical considerations when implementing the designs: for example, stopping rules or restrictions on dose skipping rules (free text).
5. Interim and final analysis information: Interim updates for trial result papers, replicability of interim and final analyses, and the information provided in the final analysis.

Data extraction from the eligible trials was conducted by Z.Y., and 18 publications (ie, 12.8% of all those included) were checked by two other authors (A.P.M. and C.Y.) for internal consistency. Disagreements were resolved through discussion to achieve consensus.

### Data Analysis

Descriptive statistics are used to summarize the characteristics of the extracted data. For continuous variables, summary statistics of median and range are displayed. For categorical variables, frequencies and percentages are displayed. To assess the trend of the number of trials implementing these six designs over time, a linear regression was fitted considering the publication year as an explanatory variable. All analyses were performed using R software version 4.0.5 (R Foundation, Vienna, Austria).^9^

### Case Studies

We replicated the results of one trial that implemented the TITE-CRM and one trial that implemented the rolling 6 design using the details provided in the respective publications. Differences between the reported trial results and our results were highlighted. In the TITE-CRM trial, DLTs were treated as a time-to-event outcome. We devised a new visualization tool using a swimmer plot to visualize the amount of toxicity information associated with the follow-up time from each participant being integrated into each interim analysis.

## RESULTS

### All Eligible Trials

**FIG 1.**
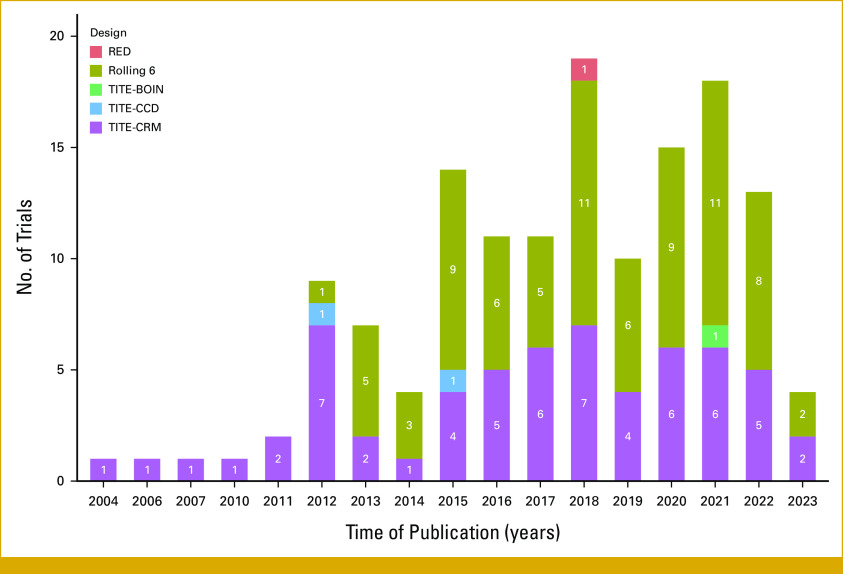
Number of dose-finding trials implementing different designs over time. Data from trials using the TITE-CRM with cycle information consideration are merged with those using the TITE-CRM, as the former is a modified version of the latter. RED, rapid enrollment design; TITE-BOIN, time-to-event Bayesian optimal interval design; TITE-CCD, TITE cumulative cohort design; TITE-CRM, TITE continuous reassessment method.

We identified a total of 141 published trials that had implemented the six mentioned designs. The majority of these trials (97.9%, 138 of 141) were cancer trials with only three (2.1%, 3 of 141) in other disease areas. Among the 141 published trials, 58 (41.1%) used TITE-CRM, 76 (53.9%) used rolling 6, two (1.4%) used TITE-CCD, three (2.1%) used TITE-CRM with cycle information, one (0.7%) used TITE-BOIN, and one (0.7%) used RED. The number of trials implementing these designs has increased over time (Fig 1) by 0.78 (with a 95% CI, 0.37 to 1.20) per year.

### Characteristics of Trials Using TITE-CRM or Rolling 6

Since the TITE-CRM and the rolling 6 design were most frequently used, we focused our subsequent results on trials implementing these two designs.

#### Basic Characteristics

**FIG 2.**
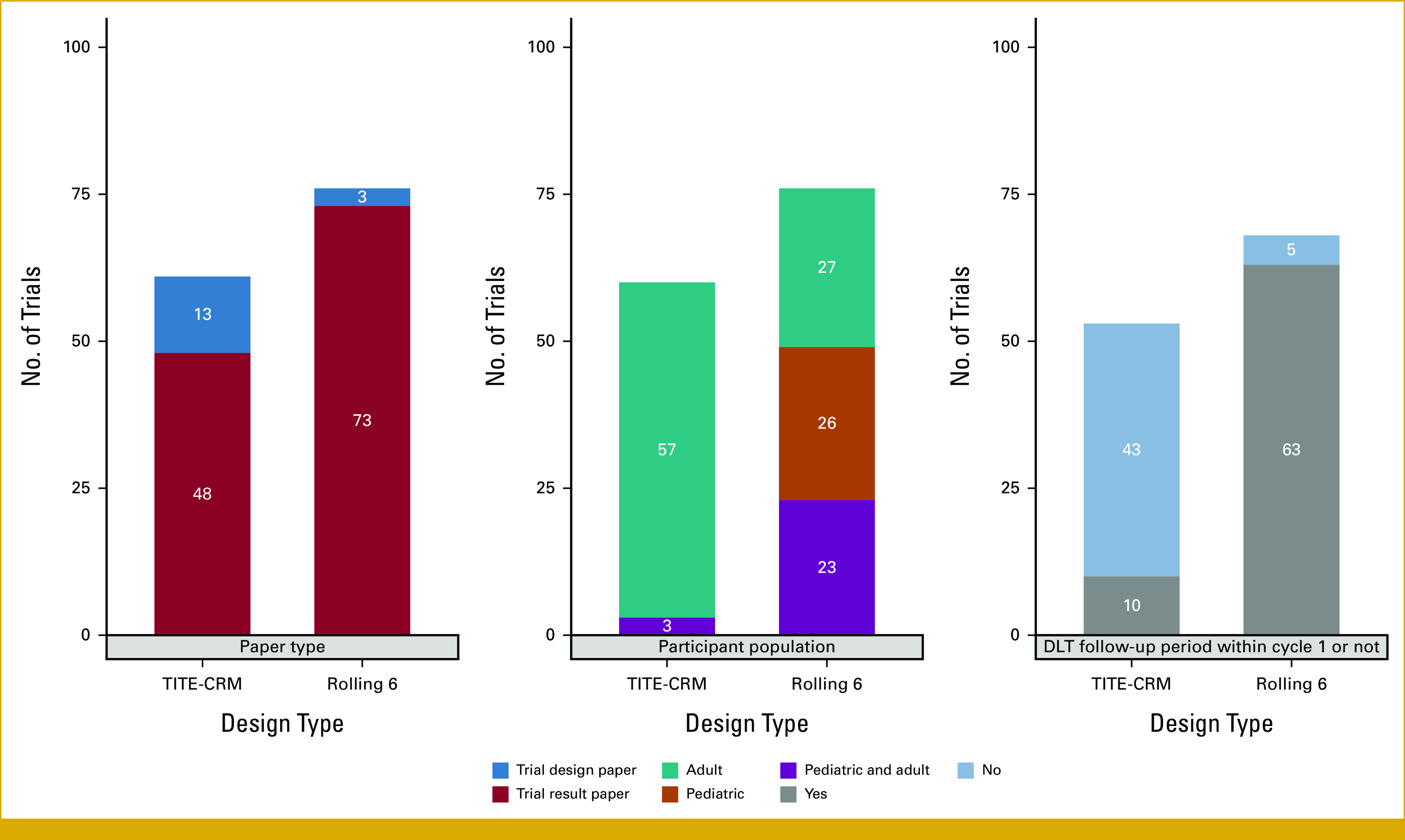
Basic characteristics of trials using TITE-CRM and rolling 6. DLT, dose-limiting toxicity; TITE-CRM, time-to-event continuous reassessment method.

Among the published trials implementing the TITE-CRM design, the majority were in adult-only populations (93.4%, 57 of 61). By contrast, the rolling 6 design was used evenly across pediatric-only populations (34.5%, 19 of 76), adult-only populations (34.2%, 26 of 76), or both (30.3%, 23 of 76). Trials using the TITE-CRM (70.5%, 43 of 61) had a DLT assessment window beyond the first cycle of treatment, compared with the rolling 6 design (6.6%, 5 of 76); see Figure 2 for the detailed comparison between these two designs. The median DLT assessment period for trials using the TITE-CRM is 12 weeks, ranging from 3 to 104 weeks.

#### Design Parameters

The TITE-CRM design utilizes a weight function to handle censored data, whereas the rolling 6 design does not require statistical models.

Among trials implementing the TITE-CRM, 54.1% (33 of 61) used the uniform weight function, assuming that a DLT had an equal chance to occur at any time point within the whole DLT assessment period. Alternative weight functions were used in 14.8% (9 of 61) of the trials. Among these trials, three used a cyclical adaptive weight function, whereas six assigned different weights to acute and chronic DLTs. Because of the sparsity of toxicities reported in the trials, it was difficult to assess whether the assumption of the weight function holds, even if the occurrence time of DLTs had been reported. Overall, most trials (57.4%, 35 of 61) did not report when DLT occurred.

The TITE-CRM design assumes a prespecified dose-toxicity model with the estimation of the model parameters continuously updated using all the data accumulated. Design parameters (including the target level of DLT, prior distribution of model parameters, skeleton probabilities) and constraints such as rules for dose (de-)escalation and early stopping need to be prespecified before the trial starts. Table 2 presents the reporting of key design parameters of published trials utilizing the TITE-CRM design. No trials stated that the DLT outcomes were to be redefined after any late-onset toxicities during the trial.

**TABLE 2.**
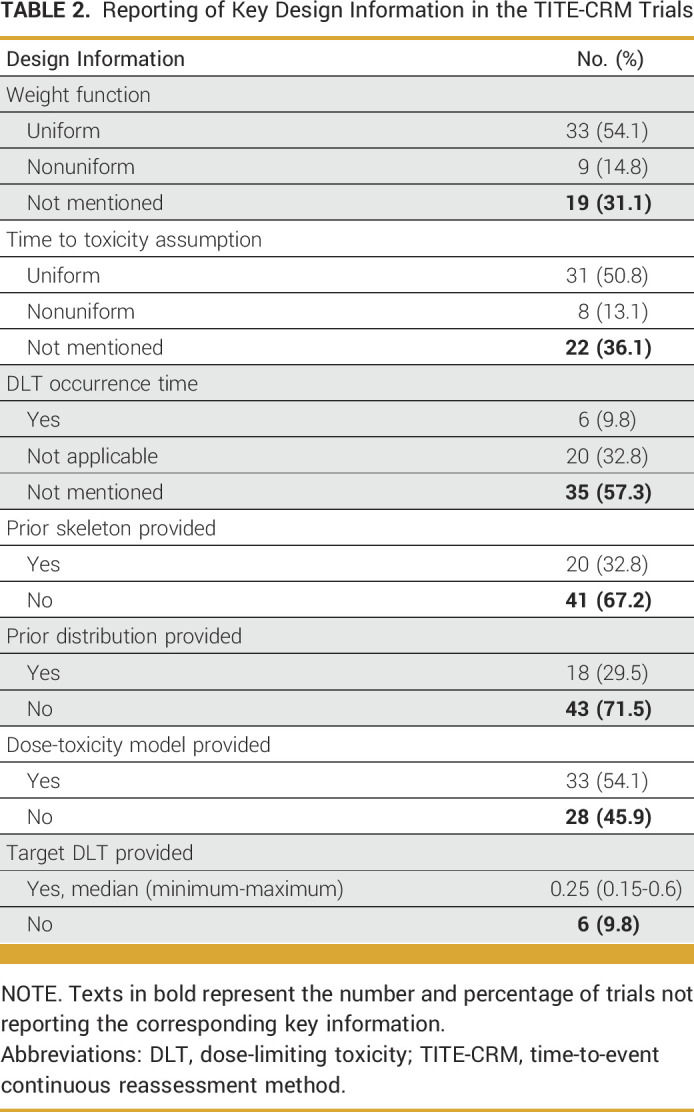
Reporting of Key Design Information in the TITE-CRM Trials

### Characteristics of Trials Using TITE-BOIN

Only one trial implementing the TITE-BOIN was identified.^10^ This was a design paper where dose decision boundaries and decision table were provided. But no result has been published yet.

### Characteristics of Trials Using TITE-CCD

Two trials implementing TITE-CCD were identified, and both were in an adult-only population.^11,12^ One trial was a phase I trial,^12^ and the other one was a phase I/II trial.^11^ Both trials used TITE-CCD with a run-in period, that is, initial escalation was in cohorts of three patients until at least one patient developed a DLT. Both trials reported the escalation and de-escalation boundaries. One trial was comprehensively reported, and the interim and final analyses were replicable. Neither of the trials reported the time to DLT occurrence.

### Characteristics of Trials Using RED

Only one phase I/II trial implementing RED^13^ was conducted in a pediatric-only population. The DLT assessment period changed from 100 to 180 days because of an increase in methotrexate duration. Time to DLT occurrence was not provided in the publication.

#### Trial Replication

For trials using the rolling 6 design, the replication of the final analysis becomes feasible on reporting the counts of DLTs and the number of participants treated under each dose level. All trial result papers provided sufficient information to facilitate replication of the final analysis, and a significant majority of trials (76.3%, 58 of 76) made their interim analysis replicable as well. By contrast, trials using the TITE-CRM require additional key information, including the design parameters, practical considerations, and DLT occurrence times, to fully replicate the trial. Only a small proportion (8.2%, 5 of 61) of trials provided all the essential model parameters necessary for replicating their final analyses.^14-18^ Among these five trials, only one trial provided the individual DLT occurrence times and participant allocation information, thus enabling the replication of the interim analysis.^14^

### Case Studies

We selected one trial that used the TITE-CRM design and another trial that used the rolling 6 design to replicate their respective interim and final analyses.

#### Case Study 1 With the TITE-CRM Design

The objective of this phase I trial of cisplatin with gemcitabine in pancreatic cancer is to determine the maximum tolerated dose (MTD) of cisplatin with gemcitabine and radiation therapy combination in patients with pancreatic cancer.^14^

The dose level of cisplatin was determined using TITE-CRM, whereas the doses for the other two therapies were held constant. Before assigning the next participant to a higher level, at least two participants had to complete at least 9 weeks of treatment at the lower level. The model recommended the level of 40mg/m2 (administered intravenously on days 1 and 15 of each chemotherapy cycle, over a 30-minute period) as the final MTD (estimated DLT rate of 0.20; observed zero DLT of five patients; acceptable toxicity rate of 0.20).

The R package bcrm (Michael Sweeting and Graham Wheeler, UK) was used to replicate the dose recommendation procedure (Data Supplement: Replicate the TITE-CRM trial; Data Supplement, Fig S3).^19^ The final analysis results related to the estimated toxicity rate (Data Supplement, Table S1) were reproducible. Specifically, a one-parameter logistic model was used to model the dose-toxicity relationship, that is, $\pi \left(d_{j},\alpha \right)=\frac{1}{1+\exp\left(-3-\exp\left(\alpha \right)d_{j}\right)}$, where α represents the dose-toxicity model parameter, with α∼Exp(1). The 95% posterior intervals and the posterior DLT estimates were calculated.

**FIG 3.**
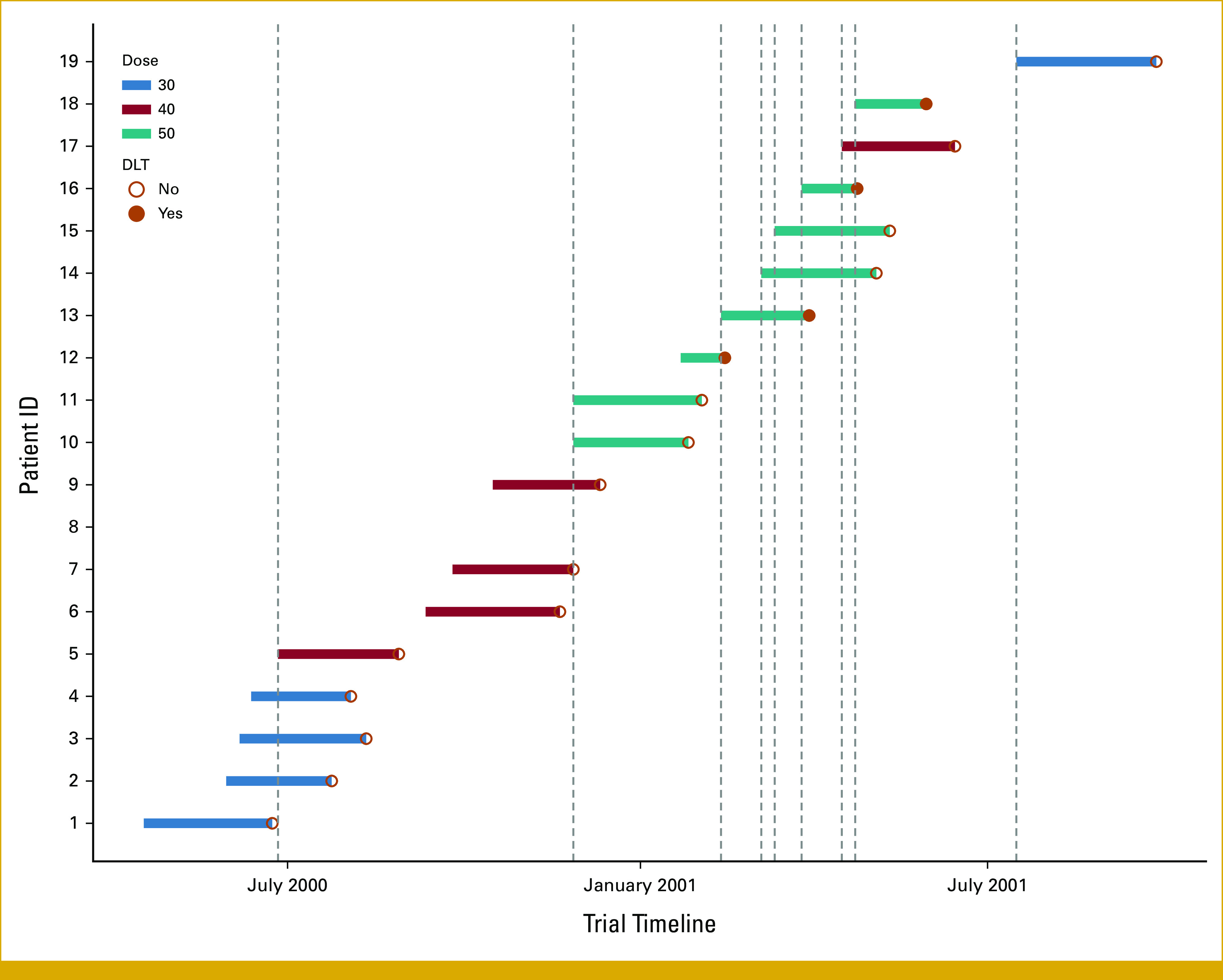
A swimmer plot illustrating dosage received, the presence of DLT, and the DLT follow-up period for evaluable participants. The vertical lines suggest when each interim analysis had been performed to assign doses for subsequent participants. DLT, dose-limiting toxicity.

Using the design parameters and escalation restrictions in the published paper, we propose the use of a more informative illustration with a swimmer plot to provide detailed information on the dose level received, DLT information, and DLT follow-up time in each interim dose decision assessment (Fig 3). The proposed swimmer plot graphically represents a participant's journey during follow-up. Each interim analysis is indicated by a vertical line, whereas the length of each horizontal bar represents the duration of an individual's DLT follow-up. Notably, when participant 5 was enrolled, only participant 1 had completed the DLT assessment period. Given the restriction that no escalation was allowed until two participants had completed toxicity follow-up, participant 5 should have been treated at dose level 1. Instead, they were escalated to dose level 2 as depicted in the trial paper. The paper did not provide an explanation for the deviation from their preplanned practical consideration. One possible reason is that they allowed for dose escalation when the equivalent of two patients' follow-up (ie, 18 weeks) was reached.

In this case study, participant 1 was enrolled on April 17, 2000, and participant 19 completed the study on September 27, 2001. The trial duration was 75.4 weeks. However, assuming that the same number of patients was to be enrolled to the trial, implementing a trial design requiring complete follow-up would have prolonged the trial duration to 162 weeks.

#### Case Study 2 With the Rolling 6 Design

This is a phase I study of 5-fluorouracil in children and young adults with recurrent ependymoma. DLT was determined after one cycle (6 weeks). The rolling 6 design proposed by Skolnik et al^4^ was used to estimate MTD.^20^ Dose escalation was permitted if none of three to six or at most one of six participants experienced a DLT at the current dose level. However, if two or more DLTs occurred, de-escalation was required. If two of two to six participants experienced a DLT, the current dose was determined to be too toxic and no escalation to a higher dosage level was allowed. Once the MTD was determined, six additional participants were enrolled to that dose level to better describe the toxicity profile of the tested agent.

DLTs were monitored during treatment cycle 1. A total of 26 participants were enrolled, three of which were not evaluable. We successfully replicated the dose assignment procedure for this trial (Data Supplement, Fig S4).

The two case studies further highlight the different levels of information needed to replicate a TITE-CRM trial and a rolling 6 trial. Reports of trials implementing TITE-CRM designs should provide comprehensive information on the clinical parameters, design parameters, practical considerations, and participant outcome in each interim data analysis to enhance transparency and reproducibility.

**FIG 4.**
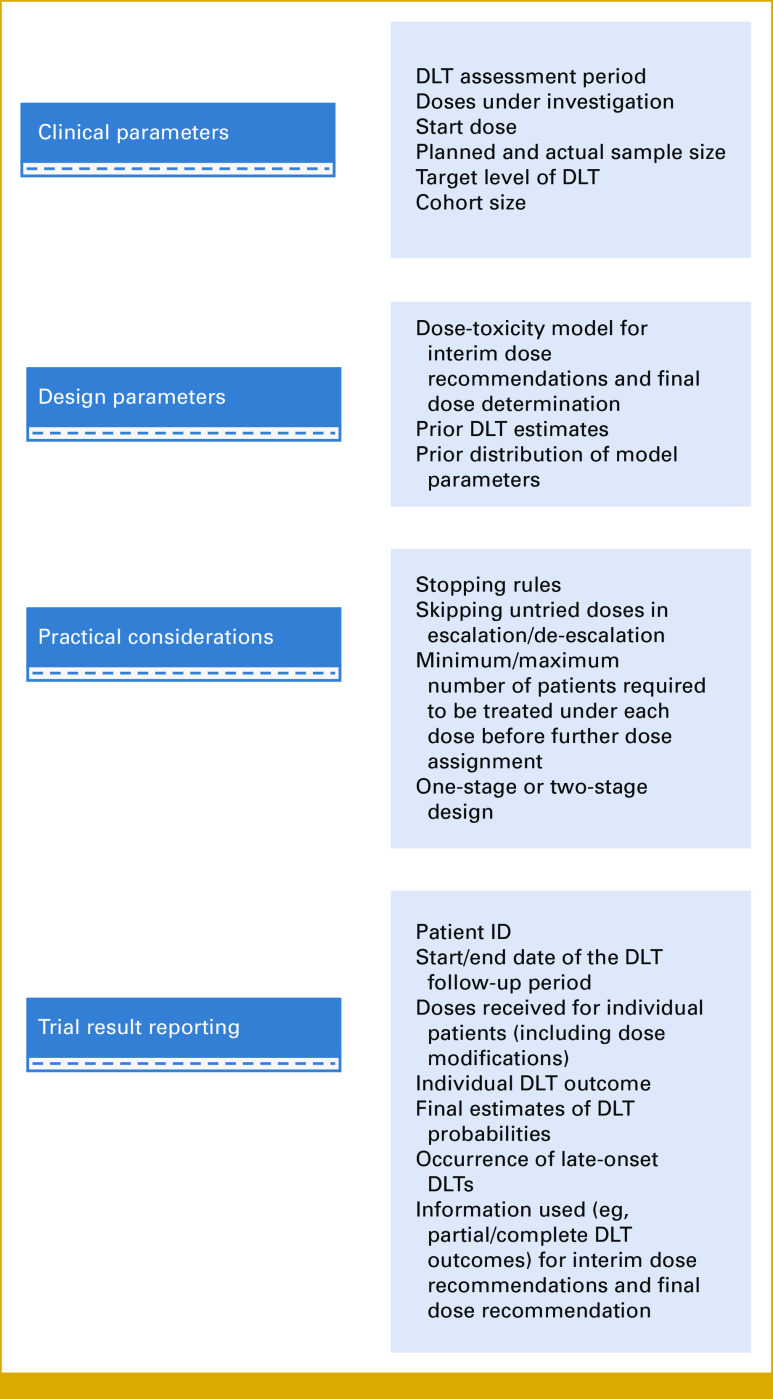
Recommendations on key elements to be provided for a trial using a TITE-CRM design (for rule-based designs, only clinical parameters and trial result reporting are required). DLT, dose-limiting toxicity; ID, identifier; TITE-CRM, time-to-event continuous reassessment method.

We provide recommendations on parameters needed when designing and reporting a trial (Fig 4).

## DISCUSSION

Late-onset toxicities can arise in early phase I oncology trials and may increase in frequency with the development of new MTAs and immunotherapies.^21^ A case study using data from bortezomib dose-finding trials indicated that 54% of the observed DLTs occurred after the first cycle of treatment.^22^ DLTs are usually assessed during the first cycle of treatment. One pragmatic way to deal with delayed toxicities is to allow the consideration of adverse events as DLTs if they occur after cycle 1 and appear to result from delayed or cumulative toxicities, as in the approach taken by Lickliter et al.^23^

The development of highly active novel investigational agents has challenged the conventional assumption that DLTs occur only in cycle 1, which forms the basis of traditional phase I designs. Designs that can model the incomplete toxicity information have been proposed, and we looked at published trials that have implemented these designs in practice. To the best of our knowledge, this is the first review on the trial characteristics and reporting quality of trials using such designs.

The main characteristics of trials implementing the rolling 6 design include a shorter DLT follow-up period, and they are more frequently used in the pediatric population. Trials using the TITE-CRM account for prior information and can model the partial and complete toxicity information available at each decision-making time point. Compared with the rolling 6 design, the TITE-CRM trials tend to have a longer DLT follow-up period and offer the flexibility to adjust the weight assigned to acute and chronic toxicities on the basis of each trial's time-to-toxicity assumption.

Although TITE-CRM is reported to be more efficient than the rolling 6 design, the increased complexity of applying this design in phase I trials comes with additional reporting demands.^24^ Our findings show that replicating analysis for TITE-CRM trials is inferior to that for the rolling 6 trials. This stems from the fact that analysis using TITE-CRM can only be replicated when all design-related parameters are transparently reported—a condition often unmet in many trials. The most common reported items in trials implementing the rolling 6 design are also well reported in the TITE-CRM trials (which include the clinical parameters such as DLT follow-up period and starting dose). There is a lack of clarity or omission of vital design parameters used in TITE-CRM trials, hindering full replication of interim and final analyses. Moreover, the individual DLT occurrence time is often not reported. While this omission does not affect replication of the rolling 6 trials, it remains crucial as it provides vital data on the toxicity profile of tested agents and can guide future trials. For trials implementing the TITE-CRM, the time to a DLT occurrence is essential not only to replicate the interim analysis but also to confirm whether the weight assumption used in the model is appropriate.

Currently, there are no recommended guidelines regarding how trial designs and analysis results should be reported in phase I dose-finding trials,^25^ but the development of the SPIRIT and CONSORT extensions for this setting is currently in progress.^26^

To facilitate the use of time-to-event designs in practice and encourage transparency, reproducibility, and accurate interpretation of results for more complex trial designs, we provided recommendations on the key information that should be reported, building on earlier recommendations for designs using complete toxicity information^27,28^ (Fig 4).

In addition, we have proposed a novel approach to capture the richness of data used in each interim analysis via an informative swimmer plot (Fig 3). When contrasted with the Data Supplement (Figs S3 and S4)—which only displays the number of DLTs and participants treated at each dose—the swimmer plot provides additional information on the individual DLT follow-up duration and data used for each interim analysis. This swimmer plot encapsulates a comprehensive view, providing important details to aid understanding and interpretation of participant flow and when and what dose decisions were made.

In certain cases, data safety monitoring committees may opt for a different dose than the model's recommended dose on the basis of an overall assessment of safety and activity data. Investigators should provide a clear rationale for such decisions and report the actual dose used to enhance the reproducibility of the results. This can assist future researchers in navigating real-world trials more effectively. It is important to note that such decisions will affect trial design characteristics, including patient allocation and time to reach the MTD.

A limitation of our study is our initial reliance on the Cited By links provided by PubMed when searching for trials implementing such designs. Given that the Cited By information is generated using data submitted by publishers and from NCBI resources such as PubMed Central, the citation search may not be comprehensive. To address this, we supplemented with an additional keyword search with filters. Another limitation is our reliance on a single database, potentially omitting some published trials implementing these designs from this review. Nevertheless, as the purpose of this study was to investigate the real-world application of these designs and assess their reporting quality, we believe that our findings should still reflect a good representation of the characteristics and reporting quality of the methodological features.

In conclusion, this review of published dose-finding trials has identified deficiencies in the reporting of crucial trial design details and analysis methods, especially in TITE-CRM trials. Such omissions hinder interpretation and replication of trial results. Although the TITE-CRM is often cited to be more efficient than the rolling 6 design,^24^ the increased complexity demands more transparency and rigorous reporting. Poorly written methods that lack rigor raise concerns about the trustworthiness of results and undermine researchers' and public confidence in these designs. By proposing an informative swimmer plot and providing recommendations on the reporting of key items, we aim to encourage transparency and reproducibility and enhance accurate interpretation of trial designs, which incorporate partial and complete toxicity information for dose decisions and their trial results.
